# Supplementary material for: Endoscopic Vacuum Therapy (EVT) versus Self-Expandable Metal Stent (SEMS) for Anastomotic Leaks after Upper Gastrointestinal Surgery: Systematic Review and Meta-Analysis
Source: Life (Basel). 2023 Jan 19;13(2):287. doi: 10.3390/life13020287 (PMC9968149; doi:10.3390/life13020287)

Supplementary Table S1 Preferred Reporting Items for Systematic Reviews and Meta-Analyses (PRISMA) checklist

Section/topic	#	Checklist item	Reported on page #
**TITLE**			
Title	1	Identify the report as a systematic review, meta-analysis, or both.	1
**ABSTRACT**			
Structured summary	2	Provide a structured summary including, as applicable: background; objectives; data sources; study eligibility criteria, participants, and interventions; study appraisal and synthesis methods; results; limitations; conclusions and implications of key findings; systematic review registration number.	2
**INTRODUCTION**			
Rationale	3	Describe the rationale for the review in the context of what is already known.	3
Objectives	4	Provide an explicit statement of questions being addressed with reference to participants, interventions, comparisons, outcomes, and study design (PICOS).	3
**METHODS**			
Protocol and registration	5	Indicate if a review protocol exists, if and where it can be accessed (e.g., Web address), and, if available, provide registration information including registration number.	4
Eligibility criteria	6	Specify study characteristics (e.g., PICOS, length of follow-up) and report characteristics (e.g., years considered, language, publication status) used as criteria for eligibility, giving rationale.	4
Information sources	7	Describe all information sources (e.g., databases with dates of coverage, contact with study authors to identify additional studies) in the search and date last searched.	4
Search	8	Present full electronic search strategy for at least one database, including any limits used, such that it could be repeated.	4
Study selection	9	State the process for selecting studies (i.e., screening, eligibility, included in systematic review, and, if applicable, included in the meta-analysis).	4
Data collection process	10	Describe method of data extraction from reports (e.g., piloted forms, independently, in duplicate) and any processes for obtaining and confirming data from investigators.	5
Data items	11	List and define all variables for which data were sought (e.g., PICOS, funding sources) and any assumptions and simplifications made.	5
Risk of bias in individual studies	12	Describe methods used for assessing risk of bias of individual studies (including specification of whether this was done at the study or outcome level), and how this information is to be used in any data synthesis.	5
Summary measures	13	State the principal summary measures (e.g., risk ratio, difference in means).	5-6

Synthesis of results	14	Describe the methods of handling data and combining results of studies, if done, including measures of consistency (e.g., I^2) for each meta-analysis.	5-6
Risk of bias across studies	15	Specify any assessment of risk of bias that may affect the cumulative evidence (e.g., publication bias, selective reporting within studies).	5
Additional analyses	16	Describe methods of additional analyses (e.g., sensitivity or subgroup analyses, meta-regression), if done, indicating which were pre-specified.	6
**RESULTS**			
Study selection	17	Give numbers of studies screened, assessed for eligibility, and included in the review, with reasons for exclusions at each stage, ideally with a flow diagram.	6
Study characteristics	18	For each study, present characteristics for which data were extracted (e.g., study size, PICOS, follow-up period) and provide the citations.	6-7
Risk of bias within studies	19	Present data on risk of bias of each study and, if available, any outcome level assessment (see item 12).	7
Results of individual studies	20	For all outcomes considered (benefits or harms), present, for each study: (a) simple summary data for each intervention group (b) effect estimates and confidence intervals, ideally with a forest plot.	Table 1,2,3
Synthesis of results	21	Present results of each meta-analysis done, including confidence intervals and measures of consistency.	7
Risk of bias across studies	22	Present results of any assessment of risk of bias across studies (see Item 15).	6-7
Additional analysis	23	Give results of additional analyses, if done (e.g., sensitivity or subgroup analyses, meta-regression [see Item 16]).	7-8
**DISCUSSION**			
Summary of evidence	24	Summarize the main findings including the strength of evidence for each main outcome; consider their relevance to key groups (e.g., healthcare providers, users, and policy makers).	8-9
Limitations	25	Discuss limitations at study and outcome level (e.g., risk of bias), and at review-level (e.g., incomplete retrieval of identified research, reporting bias).	9-10
Conclusions	26	Provide a general interpretation of the results in the context of other evidence, and implications for future research.	11
**FUNDING**			
Funding	27	Describe sources of funding for the systematic review and other support (e.g., supply of data); role of funders for the systematic review.	12

From: Moher D, Liberati A, Tetzlaff J, Altman DG, The PRISMA Group (2009). Preferred Reporting Items for Systematic Reviews and Meta-Analyses: The PRISMA Statement. PLoS Med 6(7): e1000097. doi:10.1371/journal.pmed1000097

Supplementary Table S2 Detailed search strategy for systematic review

Name of database	Time span	Search strategy
PubMed	2008- June 202	Search ("Anastomotic Leak/prevention and control"[Mesh] OR "Anastomotic Leak/surgery"[Mesh] OR "Anastomotic Leak/therapy"[Mesh]) AND "Upper Gastrointestinal Tract"[Mesh]
EMBASE	2008- June 2022	('anastomotic upper gastrointestinal leakage' OR (anastomotic AND upper AND Gastrointestinal AND ('leakage'/exp OR leakage))) AND ('treatment'/exp OR treatment)

Supplementary Table S3 Newcastle Ottawa Scale (NOS) assessment for Cohort studies. Tresholds for converting the NOS to AHRQ standards (good, fair, poor):

- **Good quality**: 3 or 4 stars in selection domain AND 1 or 2 stars in comparability domain AND 2 or 3 stars in outcome/exposure domain
- **Fair quality**: 2 stars in selection domain AND 1 or 2 stars in comparability domain AND 2 or 3 stars in outcome/exposure domain
- **Poor quality**: 0 or 1 star in selection domain OR 0 stars in comparability domain OR 0 or 1 stars in outcome/exposure domain

Studies	Selection				Comparability*	Outcome			Total stars
	1.Representativeness of the exposed cohort	2. Selection of the unexposed cohort	3.Ascertainment of exposure	4.Demonstration that outcome of interest was not present at start of study	5. Comparability of cohorts on the basis of the design or analysis	6. Assessment of outcome	7. Was follow-up long enough for outcomes to occur	8. Adequacy of follow up of cohorts	
**Berlth et al. 2018**	☆	☆	_	☆	☆	☆	☆	☆	7
**Brangewitz et al. 2013**	_	☆	☆	☆	☆	☆	☆	☆	7
**El-Sourani et al. 2022**	☆	☆	_	☆	☆	_	☆	☆	6
**Hwang et al. 2016**	☆	☆	☆	☆	☆	☆	☆	☆	8
**Mennigen et al. 2015**	☆	_	☆	_	☆	☆	☆	☆	6
**Schniewind et al. 2014**	☆	☆	_	_	☆	_	_	☆	4
**Senne et al. 2022**	☆	☆	_	☆	☆	_	☆	☆	6
**Eichelmann et al. 2021**	☆	☆	_	_	☆	_	_	_	3

* A study can be awarded a maximum of one star for each numbered item within the Selection and Exposure categories; a maximum of two stars can be given for Comparability.

Supplementary Table S4. Endoscopic details

Author	Leak etiology	Treatment	EVT type	EVT placement	EVT pressure (mmHg)	Time between EVT sessions	Stent type	Stent length	Stent diameter (mm)	Time between stent sessions
**Berlth et al.**	Esophagectomy and gastrectomy for cancer									
		EVT	Manufactured	Not defined	-125	3-5 days	-	-	-	-
		SEMS	-	-	-	-	**FC SEMS**: Baixstent OEL (Leufen Medical GmbH); Ultraflex Stent (Boston Scientific); Niti-S™ Esophageal Stent (TaeWoong Medical).	Not defined	Not defined	Not defined
**Brangewitz et al.**	Esophagectomy (benign/ malignant disease) iatrogenic perforation and Boerhaave syndrome									
		EVT	Manufactured	I/E	-125	Twice a week	-	-	-	-

		SEMS	-	-	-	-	**FCSEMS** N/A models (Medwork Medical Products and Services GmbH; Leufen Medical; MTW-Endoskopie W. Haag KG; Mandel und Rupp Medizintechnik GmbH, Germany; Boston Scientific), PS (Boston Scientific)	60-150 mm	18-30	Not defined
**El Sourani et al.**	Ivor-Lewis esophagectomy for cancer									
		EVT	Eso-SPONGE	I	-75/-125	3-5 days	-	-	-	-
		SEMS	-	-	-	-	**FCSEMS** and **PCSEMS**: Wallflex Stent (Boston Scientific)	100 mm	22–28	3 weeks
**Hwang et al.**	Esophagectomy and gastrectomy for cancer									
		EVT	Manufactured	I	-125	Twice a week	-	-	-	-
		SEMS	-	-	-	-	**FCSEMS**: Niti-S stent (Taewoong Medical); Hanaro stent (Pyeongtaek); **PCSEMS**: Bona stent (Sewoon Medical)	Not defined	Shaft: 18-20; proximal throat 26-28	4-6 weeks
**Menningen et al.**	Esophagectomy for benign and malignant disease									
		EVT	Manufactured	I	-100/-125	3-5 days	-	-	-	-
		SEMS	-	-	-	-	**FCSEMS**: OEL, Leufen Medical; **PCSEMS**: Ultraflex stent, Boston Scientific; Aixstent OEL, Leufen Medical,	80-100 mm	Shaft: 28; proximal and distal throat 28-34	4-6 weeks
**Schniewind et al.**	Esophagectomy for malignant disease									

		EVT	Eso-SPONGE	I	-70/-80	2/3 times a week	-	-	-	-
		SEMS	-	-	-	-	**FCSEMS** and **PS**	Not defined	Not defined	Not defined
**Senne et al.**	Gastrectomy for cancer									
		EVT	Eso-SPONGE	I/E	-125	3-5 days	-	-	-	-
		SEMS	-	-	-	-	**FCSEMS**: Wallflex Stent (Boston Scientific)	105 mm	23 mm	4-6 weeks
**Eichelmann et al.**	Ivor-Lewis esophagectomy for cancer									
		EVT	Manufactured	I/E	-100/-125	3-5 days	-	-	-	-
		SEMS	-	-	-	-	**FCSEMS**: OEL (Leufen Medical); **PCSEMS**: Ultraflex (Boston Scientific); Aixstent OEL, (Leufen Medical)	100 mm	Not defined	4-6 weeks

Supplementary Figure S1: Fully-Covered Self-Expandable metal stent (SEMS) for the treatment of an anastomotic leakage after Ivor-Lewis Esophagectomy

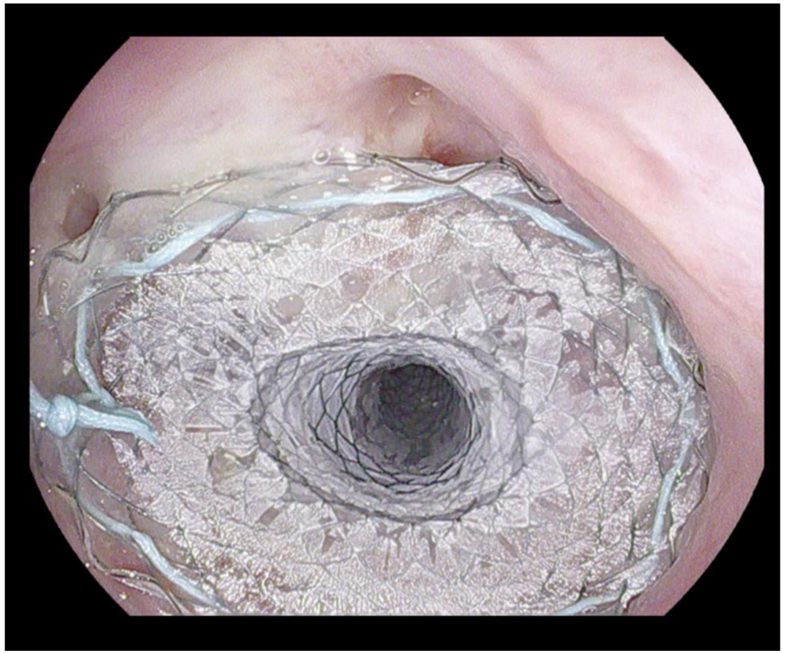

Supplementary Figure S2: Endoscopic Vacuum Therapy (EVT) technique:

A) Intracavitary placement of the sponge in a huge anastomotic dehiscence
B) Intra-luminal placement of the sponge for a small anastomotic leakage

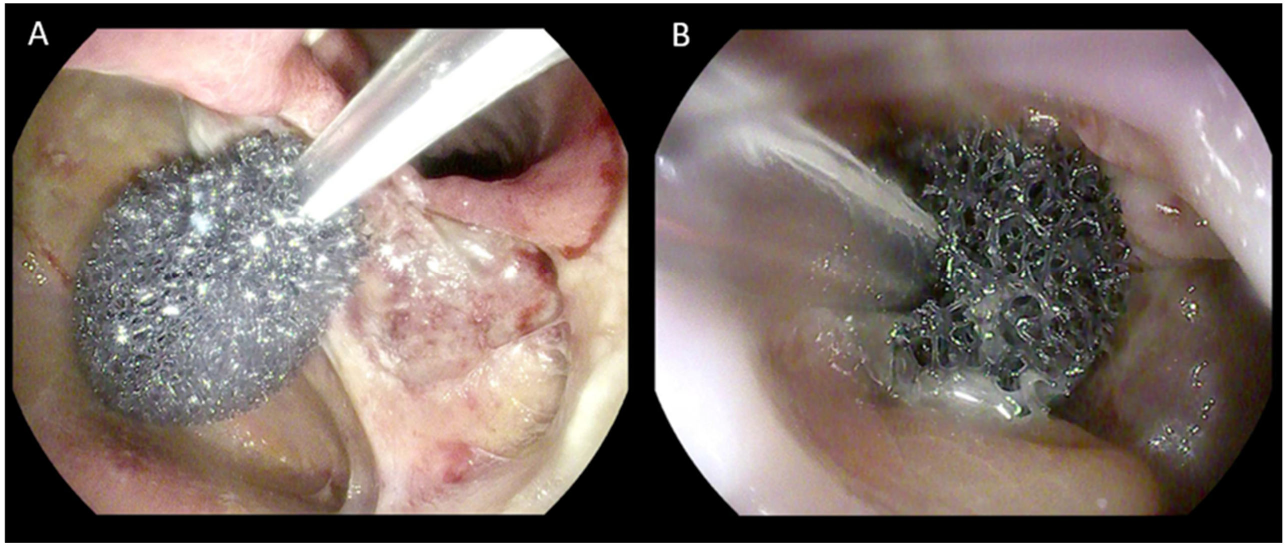

Supplementary Figure S4: Forest plot for sub-group analysis success.

Study or Subgroup	EVT Events	EVT Total	Stent Events	Stent Total	Weight	Odds Ratio M-H, Fixed, 95% CI
Berlth et al.	24	34	49	77	81.5%	1.37 [0.57, 3.28]
El-Sourani et al.	12	13	6	7	5.5%	2.00 [0.11, 37.83]
Hwang et al.	7	7	7	11	3.5%	9.00 [0.41, 198.21]
Senne et al.	8	9	5	5	9.5%	0.52 [0.02, 15.06]
**Total (95% CI)**		**63**		**100**	**100.0%**	**1.59 [0.74, 3.40]**
Total events	51		67			

Heterogeneity: $Chi^2 = 1.77$, df = 3 (P = 0.62); $I^2 = 0\%$
Test for overall effect: Z = 1.19 (P = 0.23)

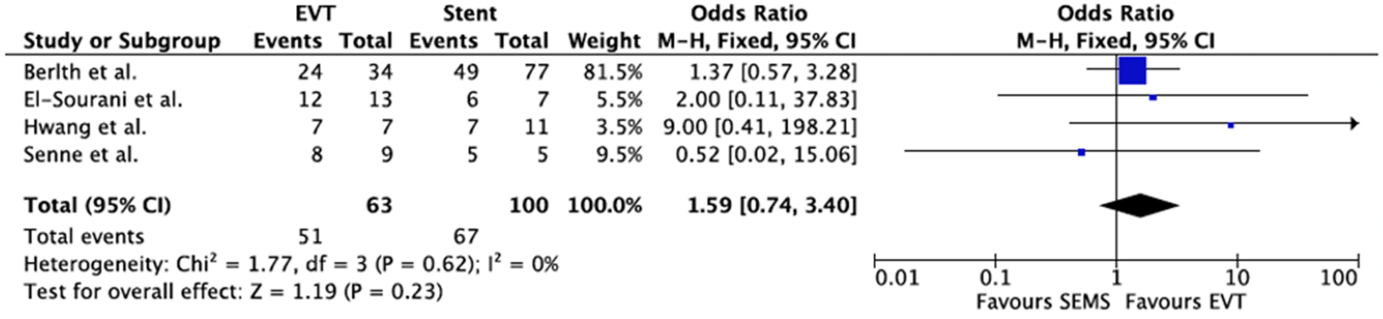

Supplementary Figure S5: Forest plot for sub-group analysis outcomes: A) Number of device B) Treatment duration C) Duration of hospitalization [21–25,28].

A)

	EVT			Stent				Mean Difference
Study or Subgroup	Mean	SD	Total	Mean	SD	Total	Weight	IV, Random, 95% CI
Berlth et al.	3	2	34	1	0	77		Not estimable
Eichelmann et al.	10	6	25	2	1	13	32.6%	8.00 [5.59, 10.41]
El-Sourani et al.	8	4	13	1	0	7		Not estimable
Hwang et al.	5	2	7	2	1	11	36.7%	3.00 [1.40, 4.60]
Senne et al.	6	4	9	2	1	5	30.7%	4.00 [1.24, 6.76]
**Total (95% CI)**			**88**			**113**	**100.0%**	**4.94 [1.84, 8.03]**

Heterogeneity: $Tau^2 = 6.13$; $Chi^2 = 11.58$, df = 2 (P = 0.003); $I^2 = 83\%$
Test for overall effect: Z = 3.13 (P = 0.002)

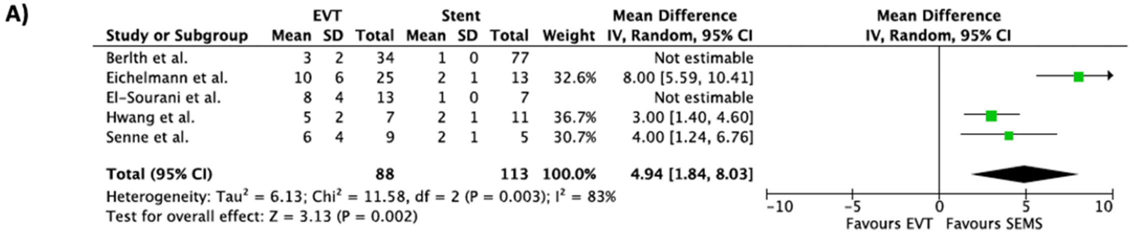

B)

	EVT			Stent				Mean Difference
Study or Subgroup	Mean	SD	Total	Mean	SD	Total	Weight	IV, Random, 95% CI
Berlth et al.	12	4	34	27	25	77	25.0%	−15.00 [−20.74, −9.26]
Eichelmann et al.	30	25	25	44	20	13	17.1%	−14.00 [−28.64, 0.64]
El-Sourani et al.	34	21	13	20	8	7	18.7%	14.00 [1.14, 26.86]
Hwang et al.	17	5	7	35	23	11	17.6%	−18.00 [−32.09, −3.91]
Senne et al.	15	10	9	26	8	5	21.7%	−11.00 [−20.58, −1.42]
**Total (95% CI)**			**88**			**113**	**100.0%**	**−9.07 [−18.99, 0.85]**

Heterogeneity: $Tau^2 = 94.03$; $Chi^2 = 17.46$, df = 4 (P = 0.002); $I^2 = 77\%$
Test for overall effect: Z = 1.79 (P = 0.07)

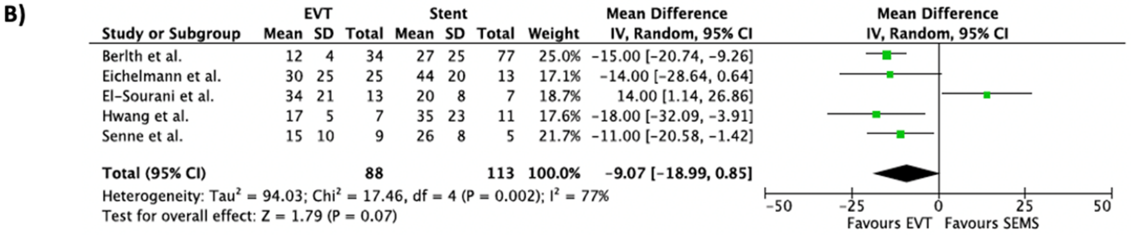

C)

	EVT			Stent				Mean Difference
Study or Subgroup	Mean	SD	Total	Mean	SD	Total	Weight	IV, Random, 95% CI
Berlth et al.	37	25	34	38	47	77	24.4%	−1.00 [−14.45, 12.45]
Eichelmann et al.	57	30	25	35	11	13	24.7%	22.00 [8.81, 35.19]
El-Sourani et al.	88	53	13	57	29	7	7.9%	31.00 [−4.94, 66.94]
Hwang et al.	54	33	7	87	87	11	3.6%	−33.00 [−89.93, 23.93]
Schniewind et al.	57	30	17	62	39	12	12.4%	−5.00 [−31.27, 21.27]
Senne et al.	38	16	9	30	5	5	27.0%	8.00 [−3.33, 19.33]
**Total (95% CI)**			**105**			**125**	**100.0%**	**7.98 [−3.39, 19.36]**

Heterogeneity: $Tau^2 = 91.17$; $Chi^2 = 10.50$, df = 5 (P = 0.06); $I^2 = 52\%$
Test for overall effect: Z = 1.38 (P = 0.17)

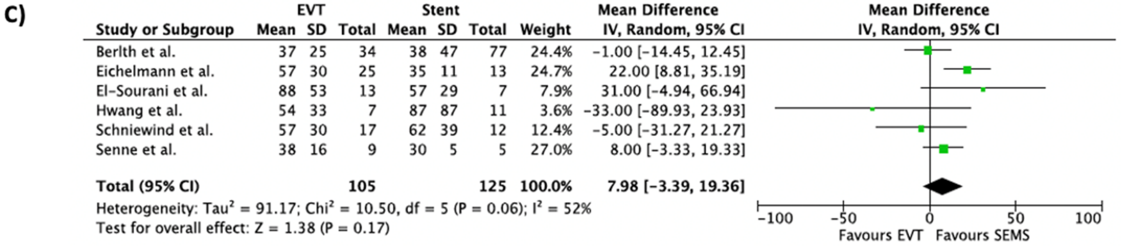

Supplementary Figure S6: Forest plot for sub-group analysis outcomes: A) Short-term complication B) Intensive Care Unit (ICU) time C) Dislocation D) Mortality [21–25,28].

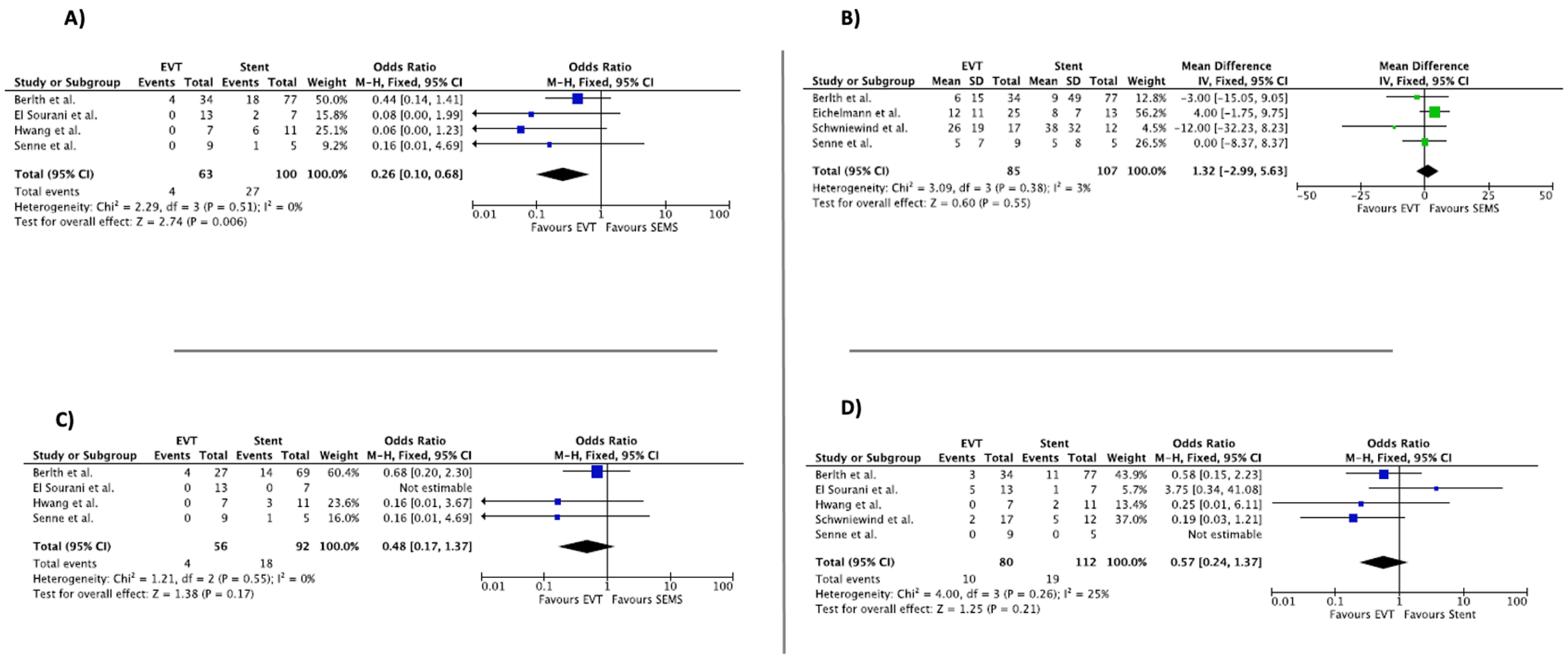

Supplement: Supplementary file 1 [file life-13-00287-s001.zip › life-2147735-supplementary.pdf]
